# Changing meaning of the rule of law

**DOI:** 10.1007/s10506-025-09468-4

**Published:** 2025-06-19

**Authors:** Jarosław Kantorowicz, Bastián González-Bustamante

**Affiliations:** 1 Leiden University, Turfmarkt 99, 2511 DP The Hague, The Netherlands; 2 Universidad Diego Portales, Santiago, Chile

**Keywords:** Rule of law, Computational social science, Word embeddings

## Abstract

Significant scholarly effort has been dedicated to defining the rule of law. The prevailing view in the literature is that the rule of law is a highly multidimensional and, as some suggest, an essentially contested concept. In this study, we employ advanced text-as-data methods, specifically diachronic word embeddings, to shed light on what the rule of law means and how its meaning has evolved over a century through parliamentary speeches in the UK and the US. We categorize the conceptualization of the rule of law into thin (procedural) and thick (substantive) definitions. Our findings indicate that procedural elements, such as rules and judiciary, maintain a strong and relatively more stable association with the rule of law. In contrast, substantive elements, which include rights and democratic principles, have become relatively less associated with the rule of law over time. Despite this decline, the rights component remains critically important to the concept, broadly equivalent in significance to procedural aspects. Because our analysis is confined to parliamentary debates from the UK and the US, the findings should be interpreted with caution when generalizing to other political contexts.

**Supplementary Information:**

The online version contains supplementary material available at https://doi.org/10.1007/s10506-025-09468-4.

## Introduction

At the surface, it may appear that the rule of law is an uncontested global norm. The ongoing clashes over it in, for instance, the EU remind us, however, that the reality is far more complicated. The rule of law —like democracy— is an “essentially contested” concept (Waldron 2002), meaning that it lacks one canonical definition. In other words, what the rule of law precisely means is not carved in stone, and, consequently, different scholars, practitioners, international organizations, and NGOs demonstrate various approaches to it. This lack of overarching consensus on what this concept means led Loughlin (2024, p. 509) to characterize the rule of law as “one of the most ambiguous slogans of contemporary public policy.” 

The proliferation of approaches and definitions of the rule of law is well exposed in two recent edited volumes on the rule of law by May and Winchester (2018) and Meierhenrich and Loughlin (2021), respectively. The legal-philosophical debates presented in these volumes shed light on how unsettled basic arguments around the rule of law are. Scholars display strikingly different conclusions regarding, among others, the origins and causes of the rule of law, the evolution of its meaning and the most influential thinkers contributing to concept building. Tense ongoing discussions furthermore revolve around the virtues of the rule of law, ranging from offering predictability to tempering arbitrary power (e.g., Krygier 2019; Raz 2019).^1^ Another example of the differences of opinion, as presented by Tamanaha (2004) and Bedner (2010), concerns the prevalence of thin and thick conceptualizations of the rule of law and when precisely the concept started including human rights (thick-substantive-conceptualization) in addition to features characterizing the quality of the legal system (thin-procedural-conceptualization). This thin vs thick debate over the rule of law is of particular importance for this paper as it stresses the idea that the rule of law is a layered concept with many discernible dimensions to track over time.

While the legal and political philosophy debates on the meaning of the rule of law are essential for the intellectual development of the field and are likely to flourish in the future, given an emerging backlash towards the rule of law in recent years, in this article, we propose a radically different approach to provide insights into the meaning and the evolution of the concept of the rule of law. We employ cutting-edge automated text analysis methods from computer science and information retrieval fields —namely, word embeddings— to understand and trace the changing meaning of the rule of law. We do so by drawing on over a hundred years of parliamentary debates in the UK House of Commons (Schwartz 2022) and the US Congress (Gentzkow et al. 2018). Our choice of parliamentary debates as data sources in this project stems from the fact that the rule of law is rarely strictly defined and codified in law or legal judgments. It is furthermore as much philosophical as political concept and, hence, we believe that examining how political elites in parliaments use (and perhaps also understand) the concept of the rule of law is the most suitable approach. For the same approach, see, for instance, Schwartz (2022).

Conceptually, word embeddings —the main method employed in this paper— rely on the assumption that the meaning of words can be uncovered through the context of surrounding words. This distributional assumption was famously paraphrased by Firth (1957, p.11), who wrote “You shall know a word by the company it keeps!”. Differently from the bag-of-words approaches to automated text analysis, which consider occurrences and frequencies of all words appearing in the text, the word embeddings are trained on smaller chunks of text (windows) to adhere to the distributional assumption. Technically speaking, word embeddings are vectors that situate words/terms in a high-dimensional vector space. Words with close meaning are located not far from each other in this vector space. The closeness in the vector space reflects, therefore, the similarity of the meaning and is measured by a specific similarity score (most typically by the cosine similarity). Of interest for this project is the closeness (similarity) of the term “rule of law” with the associated terms that capture its various thin and thick dimensions, for example, “stable laws”, “judicial independence”, and “human rights”. By comparing the closeness (similarity) of these terms over time, this article taps into the changing meaning and understanding of the rule of law. As such, this paper delivers a tool to uncover the history of the rule of law in the two jurisdictions of the common law tradition where the concept of the rule of law was arguably coined.

By now, there is a small yet growing number of path-breaking works applying word embeddings in the domain of social science to trace the changing meaning of, for instance, gender and ethnic stereotypes (Garg et al. 2018), social class (Kozlowski et al. 2019) and equality (Rodman 2020). One recent application has also investigated the changing meaning of violence (van Breen et al. 2024). In law, one notable application of word embeddings to capture the meaning is demonstrated by Schwartz (2022). This work, which largely informs the present article, uncovers the changing discourse around the UK’s constitution. Given that there is only a handful of word embedding applications in law and legal philosophy, it is important to stress that, besides providing substantive insights into the meaning and evolution of the rule of law concept, the article also makes key methodological advances in the field of empirical legal studies. Some notable exceptions to using word embeddings to support legal analysis are also works by Chalkidis and Kampas (2019) and Ash and Chen (2019).

We empirically show in this paper that substantive (rights and democracy) components of the rule of law become conceptually more distinct from the rule of law than procedural elements (especially aspects related to the quality of rules), which are relatively more stably associated with the rule of law. While declining in relation to the rule of law, the substantive rights dimension remains an important (if not the most important) aspect of the rule of law, largely on balance with procedural components.

We proceed as follows. In the next section, we demonstrate a variety of thinking and approaches towards the rule of law. This part dictates several of the operational choices we make in the analytical part of the paper, such as, especially, the adherence to the thin and thick approaches towards the rule of law. We then move on discussing the data and research design choices and continue describing the results alongside robustness checks. Lastly, we provide a short conclusion and outlook for future research.

## What is the rule of law?

Although the concept of the rule of law dates to the 19 th century and seems to be a widely accepted principle of law and state organization today, there is no scholarly consensus on what precisely the rule of law means (Gutmann et al. 2024; Gutmann and Voigt 2018; Meierhenrich and Loughlin 2021; Voigt 2012). Not only is there no single scholarly definition, but as some describe it, the rule of law is an “essentially contested” and multidimensional concept (Waldron 2002). This leads to multiple conceptual approaches in the broader rule of law scholarship. In what follows and based on Jowell (2015), we provide a brief overview of distinct approaches to the rule of law, which inform our methodological choices presented in the subsequent sections.

Albert Venn Dicey —the 19 th-century British legal philosopher and an early thinker of the modern rule of law— conceptualized the rule of law in terms of four central features: legality, certainty, equality and access to justice and rights (Dicey 1915; Jowell 2015). Such enumeration of features from early on was indicative that the rule of law is a multidimensional concept. For Dicey, one of the most important virtues of the rule of law was to protect individuals from discretionary powers wielded by officials. In his words, “no man is punishable or can be lawfully made to suffer in body or in goods except for a distinct breach of law established before the ordinary courts of the land” (Dicey 1915, p. 111). For many, Dicey’s conceptualization of the rule of law was largely procedural, without referencing the substance of law and what arguably constitutes a good law (Jowell 2015). To confirm it, scholars refer to Dicey’s famous quote, which says that “When Voltaire came to England —and Voltaire represented the feeling of this age— his predominant sentiment clearly was that he has passed out of the realm of despotism to a land where the laws might be harsh, but where men were ruled by law and not by caprice” (Dicey 1915, p. 111).

In a similar vein, Fuller (1965) procedurally describes the rule of law and legality as a state of adhering to several features concerning the quality of enacted laws. For him, the rule of law is upheld when the laws comply with a list of eight requirements specifying the quality of the laws. According to Fuller, for the rule of law to hold, the laws need to be general, clear, stable, constant, internally coherent and not require impossible, as well as being publicly available and promulgated. Raz (1979) adds to this list of procedural requirements and formal qualities of law the necessity of access to an impartial judiciary.

This by-and-large procedural approach to the rule of law presented so far tends to be severely criticized by scholars who argue that the rule of law also needs to contain substantive elements. Among them, most notably, Dworkin (1985) departs from the rule-book conception of the rule of law and insists on the conception of rights, under which legal rules contain inherent moral content. This substantive approach to the rule of law is also well reflected in the work of Bingham (2011). Similarly to Fuller, Bingham defines the rule of law through eight features. Differently from Fuller, however, Bingham also includes substantive elements in the list of features warranting the state of the rule of law. For him, the rule of law signifies accessibility of the law, law not discretion, equality before the law, the exercise of power, human rights, dispute resolution, a fair trial, and the rule of law in international order. In 2011, the Council of Europe’s Venice Commission published a document on the rule of law, in which it accepted Tom Bingham’s definition and eight ingredients, acknowledging the importance of both procedural as well substantive aspects of the rule of law (Venice Commission 2011).

The substantive and procedural dimensions of the rule of law are likewise well reflected in the United Nations’ approaches to the rule of law. When discussing the rule of law in the context of transitional justice in conflict and post-conflict societies, Kofi Annan defined the rule of law as “a principle of governance in which all persons, institutions and entities, public and private, including the state itself, are accountable to laws that are publicly promulgated, equally enforced and independently adjudicated, and which are consistent with international human rights norms and standards. It requires, as well, measures to ensure adherence to the principles of supremacy of law, equality before the law, accountability to law, fairness in the application of law, separation of powers, participation in decision-making, legal certainty, avoidance of arbitrariness and procedural and legal transparency.”^2^

In 2020, following protracted negotiations, the EU adopted Regulation 2020/2092, making EU funding disbursement conditional on addressing violations of the rule of law. Article 2(a) states that: “the rule of law refers to the Union value enshrined in Article 2 of the Treaty on European Union (TEU). It includes the principles of legality implying a transparent, accountable, democratic and pluralistic law-making process; legal certainty; prohibition of arbitrariness of the executive powers; effective judicial protection, including access to justice, by independent and impartial courts, also as regards fundamental rights; separation of powers; and non-discrimination and equality before the law.” Next to Article 2 of the TEU, this presents the first explicit definition of the rule of law by the EU and directly connects it to aspects of EU funding and economic outcomes. The EU’s approach and its accompanying frameworks indicate a preference for a thick (substantive) conceptualization of the rule of law, suggesting that mere formal (procedural) aspects of the rule of law are not sufficient in safeguarding democracy and upholding fundamental rights (Pech 2022).

The Organization for Security and Cooperation in Europe (OSCE) goes even further in its substantive approach to the rule of law. In its 1990 Copenhagen document, the OSCE specifies that “Democracy is an inherent element of the rule of law”. Beyond that, the OSCE says that “the rule of law... does not mean merely a formal legality which assures regularity and consistency in the achievement and enforcement of democratic order, but justice based on the recognition and full acceptance of the supreme value of the human personality and guaranteed by institutions providing a framework for its fullest expression.” Although OSCE and other international organizations tend to conflate the rule of law and democracy, some argue that this may constitute a problem for deeper analyses. In fact, for some, democracy and the rule of law are independent concepts, both of which can exist without the presence of the other (Mukand and Rodrik 2020; Zakaria 1997).

A distinct approach, albeit also substantive in nature, is likewise propagated in the economic literature investigating the relationship between the rule of law and socio-economic performance via cross-country regressions (e.g. Acemoglu et al. 2001; Haggard and Tiede 2011; Haggard et al. 2008). As such, in economics, the rule of law is often equated with the protection of property rights and the lack of (civil) conflict, further expanding our substantive understanding of the rule of law.

Based on the discussion so far, it is evident that the rule of law is a multidimensional concept with different actors and scholars stressing different procedural and substantive elements of the rule of law as most important for the concept. In this article, we stay agnostic towards what constitutes the ideal definition of the rule of law. Instead, we acknowledge that the rule of law is a multidimensional concept and empirically analyze how the various components and features were and are related to the rule of law historically and contemporaneously. To this end, we make a clear distinction between components and features related to a thin (procedural) conceptualization (Fuller 1965; Tamanaha 2004) and a thick (substantive) conceptualization of the rule of law (Bedner 2010; Bingham 2011). Within the procedural components, we further distinguish the features linked to the quality of the rules and the legal system more broadly from those related to judiciary and judicial system aspects. As to substantive elements, we divide them into elements connected to rights and democracy. This leaves us with four conceptual dimensions to trace over time: (1) procedural rules; (2) procedural judiciary; (3) substantive rights; and, lastly, (4) substantive democracy attributes. The following section discusses how we operationalize and measure these dimensions.

## Data and methods

Table 1 demonstrates the list of n-grams (terms) chosen to capture each of the four dimensions (attributes) of the rule of law.^3^ The list was created via an iterative process whereby ten experts were consulted about the initial choice of n-grams. The final list was tailored based on the insights from the expert. The terms chosen for the procedural rules dimension were primarily selected based on Lon Fuller’s conceptualization of the rule of law, who derived a list of eight qualities of laws. In our case, the list is slightly longer as it also covers close synonyms as well as nouns and adjective versions of terms. The terms related to the procedural judiciary dimension are frequent n-grams, which relate to the qualities of the judiciary. The substantive rights dimension is captured by frequently occurring n-grams, including the rights term. Lastly, it has been decided that only two terms, democracy and democratic, will capture the substantive democracy dimension. Such a parsimonious approach was opted for given that these two terms are encompassing and arguably frequently used in parliamentary speeches.

**Table 1:** The rule of law attributes (dimensions) and the corresponding n-grams

Procedural		Substantive	
Rules	Judiciary	Democracy	Rights
clarity	judicial_impartiality	democracy	civil_liberties
clear	judicial_independence	democratic	civil_liberty
stability	judicial_protection		civil_rights
stable	judicial_accountability		civil_right
constancy	judicial_autonomy		human_rights
constant	judicial_review		human_right
certainty	constitutional_review		fundamental_rights
certain			fundamental_right
predictability			constitutional_rights
predictable			constitutional_right
generality			property_rights
general			property_right
universalism			minority_rights
universal			minority_right
universality			individual_rights
prospectivity			individual_right
prospective			procedural_rights
retroactivity			procedural_right
retroactive			inalienable_rights
publicity			inalienable_right
public			
publicly			
transparency			
transparent			
congruence			
congruent			
consistency			
consistent			
coherence			
coherent			

Our empirical strategy targets the analysis of concept drift in textual corpora by relying on diachronic word embedding (vector) models (Mikolov et al. 2013a, b), specifically a chronological word2vec implementation adapted from Rodman (2020) and Schwartz (2022). These models allow us to map the semantic relationships (associations) between the rule of law and its different dimensions over time, providing insights into how the concept evolves. The word2vec approach builds a highly dimensional language representation space where similar words are closely “embedded” (located), and similarity is derived from the contexts in which those words appear. This distributional assumption (Firth 1957, p.11) contrasts with bag-of-words approaches (e.g., topic models) that focus on co-occurrence rather than direct contextual relationships.

While other methods —such as dynamic embeddings or GloVe— also offer interesting avenues for detecting semantic shifts, we reasoned that diachronic word2vec provides a well-established, interpretable, and computationally efficient framework for tracking concept drift over considerable periods. Nevertheless, to bolster confidence in our findings and account for different modeling perspectives, we also implement additional approaches, including a DistilBERT model, detailed below. Researchers can thus consider an array of methodologies, adapting their choice depending on data size, linguistic variance, and the specific research questions at hand.

For training the models in the UK, we rely on the same Hansard data used by Schwartz (2022). This dataset comprises 11,160 parliamentary speeches and almost 200 million words from both the House of Commons and Lords between 1805 and 2020. However, it does not entirely cover Hansard since it focuses on politico-legal topics such as the constitution, parliamentary sovereignty, separation of powers, the rule of law, devolution, human rights, democracy, civil liberties, judicial independence, judicial review, ministerial accountability, among others.

For the US, we use Gentzkow et al. (2018) data, also used by Rodriguez et al. (2023). This dataset contains congressional records from 1873 to 2017, which, after matching the sample with the UK topics, comprises of 411,361 utterances and around 317 million words.^4^ The complete US corpus contains around 16.7 million speeches and almost 1.7 billion words for the whole period. It is important to note that in total, we use three corpora: (1) Hansard-focused corpus based on Schwartz (2022); (2) the US-focused corpus that matches Hansard data; and, lastly, (3) the US entire corpus (Gentzkow et al. 2018). For the basic information on the size of the three employed corpora, see Table 2. Note that, besides the first period for the UK, the corpora are broken down into slices covering 25 years.

**Table 2:** Corpora used in the analysis

Period	UK focused corpus		US focused corpus		US entire corpus	
	Speeches	Words	Speeches	Words	Speeches	Words
1800-1849	158	2,888,710	–	–	–	–
1850-1874	123	1,524,256	1,775	1,791,336	82,799	8,677,547
1875-1899	112	1,891,564	37,199	35,747,873	2,112,984	164,563,547
1900-1924	330	7,243,479	48,993	36,206,174	3,482,626	233,594,024
1925-1949	492	9,843,951	54,436	36,953,122	3,162,706	228,710,118
1950-1974	1,304	27,479,185	94,837	66,839,845	3,554,958	391,297,594
1975-1999	3,786	67,748,861	124,364	95,211,431	3,393,958	482,847,090
2000-2024	4,855	81,362,879	49,757	44,616,985	976,348	182,412,463
Total	11,160	199,982,885	411,361	317,366,766	16,766,379	1,692,102,383

Prior to employing word embedding models, we applied a number of standard pre-processing steps, such as tokenization, lowercase conversion, and removed punctuation, symbols, numbers and spaces. We also perform language stabilization. Namely, we applied 207 recodifications for relevant multiword collocations and unique concepts, and 15 recodifications were used to correct some relevant misspelt words. These recodifications are especially important for n-grams capturing the central concept of interest, i.e., the rule of law (rule_of_law), and its attributes, e.g., judicial independence (judicial_independence) and human rights (human_rights).

The chronologically trained word embedding models use word2vec with Skip-Gram with Negative Sampling (SGNS) algorithm as their loss function. We begin by fitting a comprehensive model for each country’s entire corpus, setting the dimensionality of vectors to 300. This initial model was trained for five epochs, pruning words that occur below a frequency threshold of 10 occurrences and discarding noisy words at the same threshold. We used context windows of size 10 for the training process to capture the surrounding context effectively.^5^ After fitting this initial model, we trained separate models for each chronological slice or period. For each new slice, we initialize its word vectors using those from the preceding slice, assuming that semantic relationships established in one period carry over into the next (Rodman 2020). This process was repeated for each time slice, retaining almost the same hyperparameter settings as the initial model while performing 30 bootstrap iterations per slice to estimate uncertainty measures (see Antoniak and Mimno 2018 for a similar bootstrapping approach). In the case of the UK, the first slice begins with data from 1800-1849, and for the US, from 1850-1874. These initial slices draw on vectors from the entire corpus to provide an anchor for semantic continuity and linkages across time.

In addition to these baseline models, we also fitted several SGNS word2vec variants incorporating a popularity-bias reduction strategy (Steck et al. 2024). This involved adjusting the negative sampling distribution to downweigh commonplace words and reduce their skewing effect on the learned embeddings. By doing so, we aim to mitigate popularity bias —where very common words can disproportionately influence the model— and more accurately capture the evolving semantic relationships of “rule_of_law” and its attributes. These bias-reduced models followed the same chronological and bootstrapping procedure but included parameter modifications such as subsampling at 0.001 to control for frequent words effectively and smoothing the distribution of commonplace words using a proportion of 3/4 in the negative sampling exponent following some recommendations by Steck et al. (2024).

After training, we obtain cosine similarity estimates for the n-gram “rule_of_law” and the group of attributes of the rule of law, as identified in Table 1. Cosine similarity is a standard distance metric for analyzing semantic proximity within a multidimensional embedding space. The bootstrap procedure mentioned above involves resampling the sentences in the parliamentary speeches in order to calculate mean cosine similarity values and standard deviations. We then form confidence intervals by dividing the standard deviation of each similarity estimate by the square root of the number of bootstraps, yielding a standard error that is multiplied by the appropriate z-value for a 95% confidence interval. This methodology applies both to the baseline models and the bias-reduced models, allowing us to gauge the robustness of our results and to compare how different modeling choices influence the semantic portrayal of the rule of law across time, taking into account limitations of measures such as cosine similarity (Steck et al. 2024).

We coducted two other models as robustness checks. First, we run chronologically trained word embeddings for a shorter period, namely, we start the analysis after World War I. Second, we employ DistilBERT, a transformer model pre-trained in an unsupervised fashion using the Bidirectional Encoder Representations from Transformers (BERT) model as a baseline to obtain distillation loss in terms of the same probabilities of the BERT base model and cosine embedding loss to emulate their hidden states but faster (Sanh et al. 2020).^6^ Another robustness checks consider the application of somewhat more extensive list of terms capturing the corresponding dimensions of the rule of law (see Table A1) in the Supplementary Material.

In what follows, we present the results for three corpora on how the rule of law and its derived dimensions relate to each other over more than hundreds of years.

## Results

**Fig. 1:**
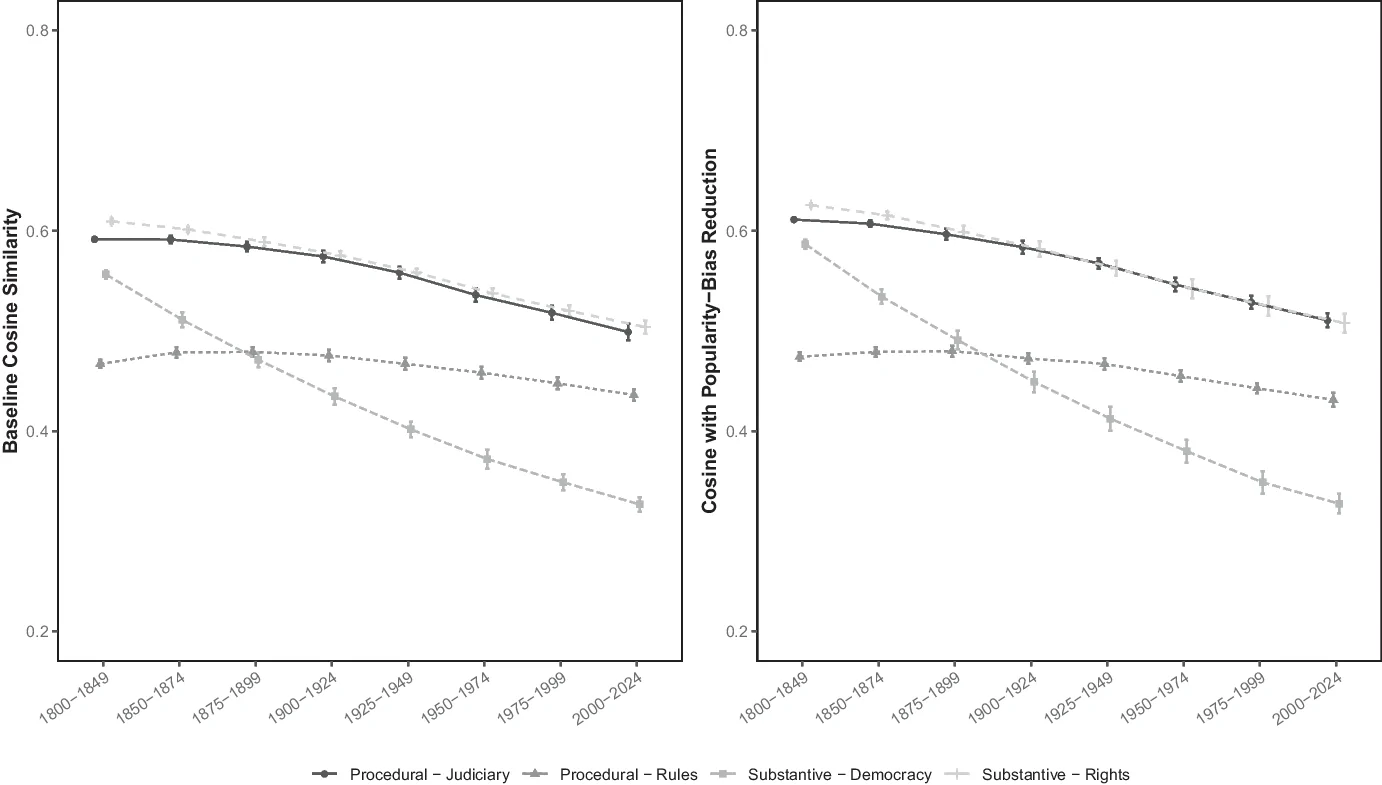
The changing concept of the rule of law in the UK (focused corpus)

**Fig. 2:**
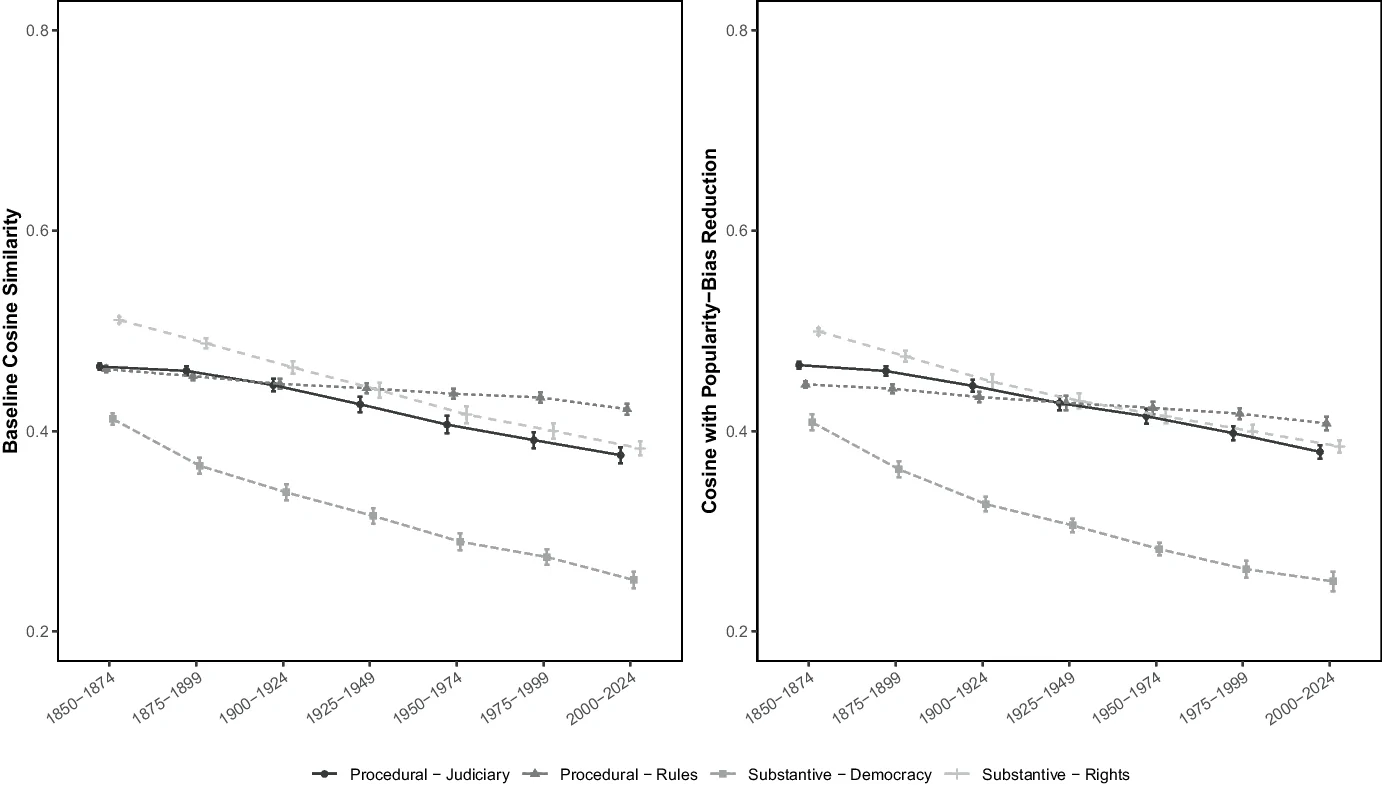
The changing concept of the rule of law in the US (focused corpus)

**Fig. 3:**
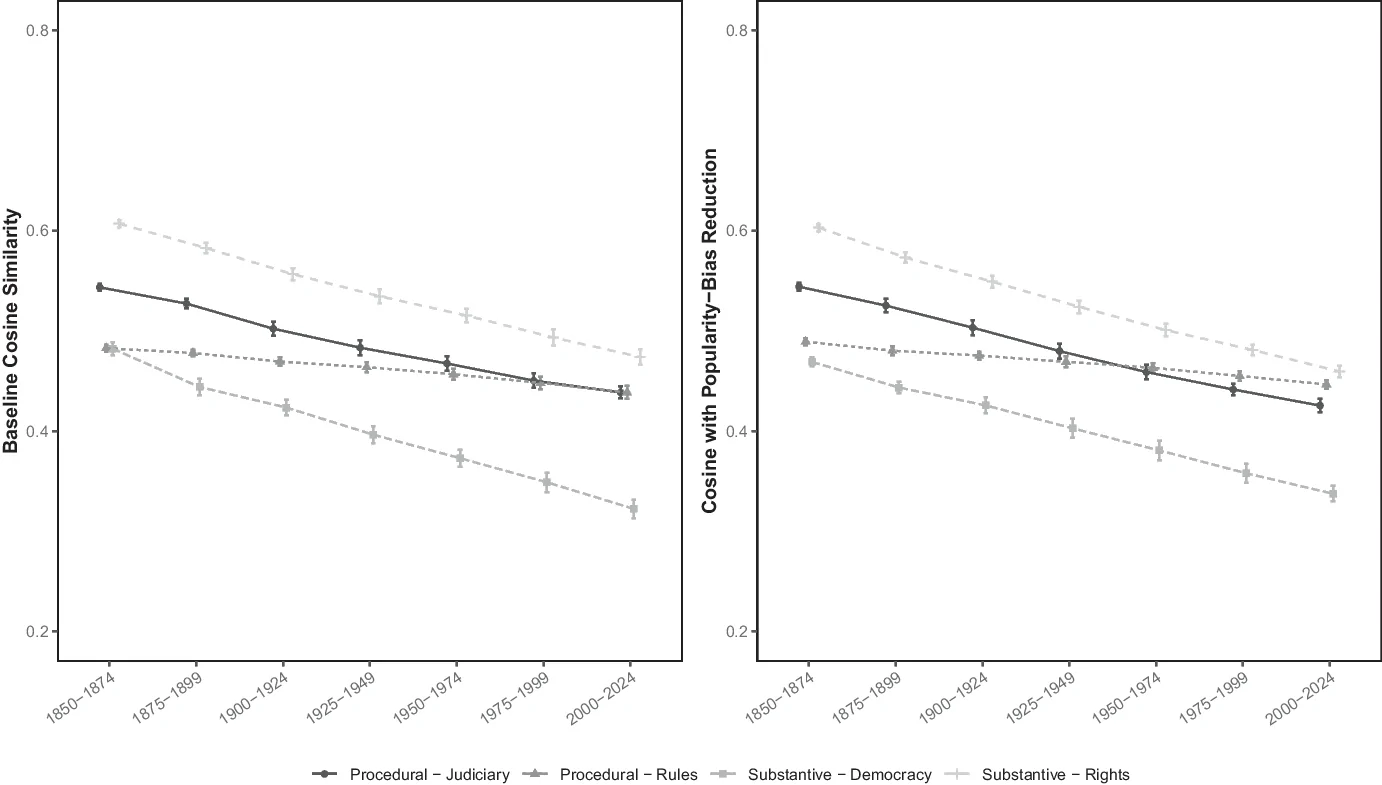
The changing concept of the rule of law in the US (entire corpus)

Figures 1, 2 and 3 show the main results, namely aggregated, baseline cosine similarity scores (their bootstrapped means and confidence intervals) as well as the scores with popularity-bias reduction for each identified dimension of the rule of law for the UK (focused sample) and two US (focused and entire) samples, respectively. The cosine similarities capture the association between the rule of law concept and its four dimensions. Thus, the higher the score, the more the rule of law is associated with the given dimension. Although we privilege the interpretation of baseline measures, the correlation between the baseline and bias-reduction scores is relatively high.

As for the UK (Figure 1), we observe that in the earliest periods under investigation, substantive elements (the rights dimension in particular) and procedural judiciary dimension were somewhat more related to the rule of law than the procedural component associated with the quality of rules. Of note is a subsequent gradual decline of cosine similarity for all dimensions of the rule of law over time, signifying that the linguistic context in which the rule of law itself is used is increasingly different from the context in which the other terms are used. It needs to be stressed, however, that in relative terms, the decline in cosine similarity for the substantive elements (rights and democracy) is greater than the decline observed for the procedural components (especially the rules dimension). Focusing on the latest period captured by the data (2000-2024), one should further note that the rights dimension tends to be the most associated with the rule of law and is followed by procedural components, i.e., judiciary and rules, respectively. The democracy dimension currently seems to be the aspect the least associated with the rule of law. One could, therefore, argue that democracy gains conceptual distinction from the rule of law.

The results for the US based on the matched corpus (Figure 2) are by and large like the results for the UK. The general trend of the substantive features of the rule of law departing more strongly from the concept of the rule of law than procedural components is upheld. Some minor differences relate to the ordering of strength of association between the four dimensions and the rule of law over time. In the early periods, the rights dimension was the most associated with the rule of law, followed by judiciary and procedural component related to rules. The least related to the rule of law was the aspect linked to democracy. Contemporarily, the rules dimension seems more related to the rule of law than the rights aspect. These two most associative dimensions are followed by the judiciary component and democracy. One should note, however, that, like for the UK, the democracy feature has become the most distinct over time.

The analysis for the US based on the entire corpus (Figure 3) generally shows the same trends as the analysis based on the matched (focused) corpus. One important difference, however, relates to the observation that in the latest period (2000-2024), we discern slightly different ordering of dimensions most associated with the rule of law. It is evident that the rights aspects maintained its position as the most prevalent, followed by procedural features of rules and judiciary. This result is very much in line with the result obtained for the UK, increasing our confidence in the overall analysis. This also enhances our confidence in general observations one can make. Namely, the procedural elements of the rule of law, particularly the dimension of quality of rules, are relatively more strongly associated with the rule of law than the substantive components. While declining in relation to the rule of law, the substantive rights dimension nonetheless remains an important aspect of the rule of law, largely on par with the procedural elements.

A number of results based on alternative modelling techniques (chronologically trained word embeddings based on a shorter period and cosine similarities based on an alternative list of keywords), which we show in Supplementary Material, tend to generally support these findings. The results from the DistilBERT model are somewhat less conclusive as they provide for much less variation across identified rule of law dimensions. In addition, we have evaluated our models using WordSim-353, which provides a gold standard based on a set of words labeled by human coders with similarity values to compare and correlate our model results with human similarity judgments (Finkelstein et al. 2002; Jatnika et al. 2019). The models used to initialize the chronological training process showed values of 0.525 for the UK, 0.580 for the US, and 0.622 for the US entire corpus. It is relevant to note that the average performance on this test is around 0.580, and only a few applications show higher performance (e.g., Salle and Villavicencio 2018; Song et al. 2018). We also find substantively similar results if we consider a more extensive list of n-grams capturing the corresponding dimensions of the rule of law (see Figures A6, A7 and A8 in the Supplementary Material).

## Conclusions and outlook

While drawing on computational social science methods, this article contributes to major ongoing legal, political and philosophical debates regarding the prevalence of different dimensions and attributes of the rule of law. It provides an objective manner to capture the concept of the rule of law and its various components. Against this backdrop, describing the methodology as “objective” refers to its replicable and systematic nature: measuring and comparing (cosine) similarity across word embeddings trained on parliamentary speeches in the UK and the US over hundreds of years. However, it is important to acknowledge that the underlying tools can introduce bias and that objectivity here does not imply an absence of interpretive or methodological assumptions. Indeed, even though we offer a principled way to capture how the concept of the rule of law and its different dimensions change over time, our computational measures, such as cosine similarity, are a product of both algorithmic design as well as the particularities of the corpora and terms selected.

We demonstrate that the substantive aspects of the rule of law, such as rights and democracy, are becoming increasingly distinct from the concept of the rule of law compared to procedural elements, which include the quality of rules and maintaining a more consistent association with the rule of law. Although the importance of substantive rights in relation to the rule of law is decreasing, they continue to be a crucial, and possibly the most crucial, part of the rule of law, roughly equivalent in importance to procedural components.

Our study is primarily descriptive, aiming to delineate the rule of law and trace its evolution over time. From a normative perspective, there might be a temptation to advocate for greater conceptual clarity and clearly differentiate the rule of law from other fundamental concepts. Notably, the concept of democracy has increasingly been recognized as distinct over time. It raises the question: Will the aspects of substantive rights similarly evolve to become more distinct in the future? Conceptually, the persistence of this trend would be beneficial, as blending concepts can undermine their analytical precision (Craig 2017). Viewing the rule of law primarily as a procedural dimension, with the aspects of rights and democracy standing independently, could enhance the clarity of theoretical frameworks and facilitate more straightforward empirical investigations.

Beyond this normative point, future work in this domain could entail measuring the meaning and understanding of the rule of law across a wider array of jurisdictions and contexts, rather than limiting the analysis to US and UK corpora. While our focus on these two Anglophone datasets allows for a descriptive examination of concept drift, it inevitably represents a clear limitation of our study’s broader validity. Expanding the scope to include texts from continental Europe, BRICS countries, and other regions with different legal traditions, languages, and governance structures would permit a more comprehensive view of how the rule of law evolves globally.

Another limitation follows from using parliamentary debates to map a legal concept. Although the rule of law is a multidimensional construct debated not only in parliaments but also in academic research, governmental white papers and international commission reports, our approach relies on parliamentary speeches. It thus cannot capture the full diversity of perspectives. While this focus affords an extended historical comparison, it necessarily reduces the concept’s multidimensionality by emphasizing the discourse of political elites. Broader datasets encompassing formal government or international reports could yield richer insights and help refine the empirical mapping of the rule of law.

Nevertheless, the approach we propose, based on estimating, contrasting and discussing scores from word embeddings, could be applied to a broader variety of textual sources and content. One could also provide a principled way to derive the weights for the rule of law indicators and enable a re-evaluation of existing country rankings in terms of adherence to the rule of law principles. The innovation in this aspect could come from applying similarity scores of word embeddings (cosine similarities) as weights assigned to constitutive parts of the rule of law indicators. Such an approach could extend to other composite indicators, not least democracy. Initial research efforts in this direction have been presented by González-Bustamante and Kantorowicz (2024). Finally, a promising future extension would be to examine judicial decisions, thereby illustrating how courts contribute to the evolving definition and understanding of the rule of law.

## Supplementary Information

Below is the link to the electronic supplementary material.

Supplementary file 1 (pdf 1851 KB)

## Footnotes

1 For a comprehensive overview of the academic field of rule of law, see Kantorowicz and González-Bustamante (2024).

2 See https://docs.un.org/en/S/2004/616 (last accessed on June 6, 2025).

3 The data, code and supplementary material are deposited on  GitHub and Zenodo https://github.com/RoL-project/unravelling-rule-of-law.

4 Hansard’s data used by Schwartz (2022) was already filtered as was mentioned. We replicate the same sampling process during our pre-processing stage for the US data. The complete list of concepts is as follows: constitution, civil liberties, constitutional convention, federalism, fundamental rights, human rights, independence of the judiciary, judicial independence, judicial power, judicial review, accountability, democracy, sovereignty, prerogative, separation of powers and the rule of law.

5 We also used Cython dependency to speed up the training process using C++ and 12 cores of CPU.

6 A number of works have used transformer models from the BERT family, such as some distilled or fine-tuned versions, to classify political content and speech (Timoneda and Vallejo Vera 2024).
