# Supplementary material for: Changing meaning of the rule of law
Source: Artif Intell Law (Dordr). 2025 Jun 19;34(4):1209–24. doi: 10.1007/s10506-025-09468-4 (PMC13743594; doi:10.1007/s10506-025-09468-4)

Changing Meaning of the Rule of Law

Supplementary Information File

Abstract

Significant scholarly effort has been dedicated to defining the rule of law. The prevailing view in the literature is that the rule of law is a highly multidimensional and, as some suggest, an essentially contested concept. In this study, we employ advanced text-as-data methods, specifically diachronic word embeddings, to shed light on what the rule of law means and how its meaning has evolved over a century through parliamentary speeches in the UK and the US. We categorize the conceptualization of the rule of law into thin (procedural) and thick (substantive) definitions. Our findings indicate that procedural elements, such as rules and judiciary, maintain a strong and relatively more stable association with the rule of law. In contrast, substantive elements, which include rights and democratic principles, have become relatively less associated with the rule of law over time. Despite this decline, the rights component remains critically important to the concept, broadly equivalent in significance to procedural aspects.

Keywords: rule of law; computational social science; word embeddings

Table A1: The rule of law attributes (dimensions) and the corresponding n-grams – Alternative list

Procedural		Substantive	
Rules	Judiciary	Democracy	Rights
clarity	judicial_impartiality	democracy	civil_liberties
clear	judicial_independence	democratic	civil_liberty
stability	judicial_protection	separation_of_powers	civil_rights
stable	judicial_accountability	checks_and_balances	civil_right
constancy	judicial_autonomy	check_and_balance	human_rights
constant	judicial_review		human_right
certainty	constitutional_review		fundamental_rights
certain	access_to_justice		fundamental_right
predictability			constitutional_rights
predictable			constitutional_right
generality			property_rights
general			property_right
universalism			minority_rights
universal			minority_right
universality			individual_rights
prospectivity			individual_right
prospective			procedural_rights
retroactivity			procedural_right
retroactive			inalienable_rights
publicity			inalienable_right
public			
publicly			
transparency			
transparent			
congruence			
congruent			
consistency			
consistent			
coherence			
coherent			
accountability			
accountable			
proportionality			
proportional			
efficiency			
efficient			
rationality			
rational			
logical			
practicability			
practical			
legitimacy			
legitimate			
procedural_justice			
due_process			
open_government			
morality			
moral			
professionalism			
professional			

Figure A1: The changing concept of the rule of law in the UK – Alternative chronologically trained models

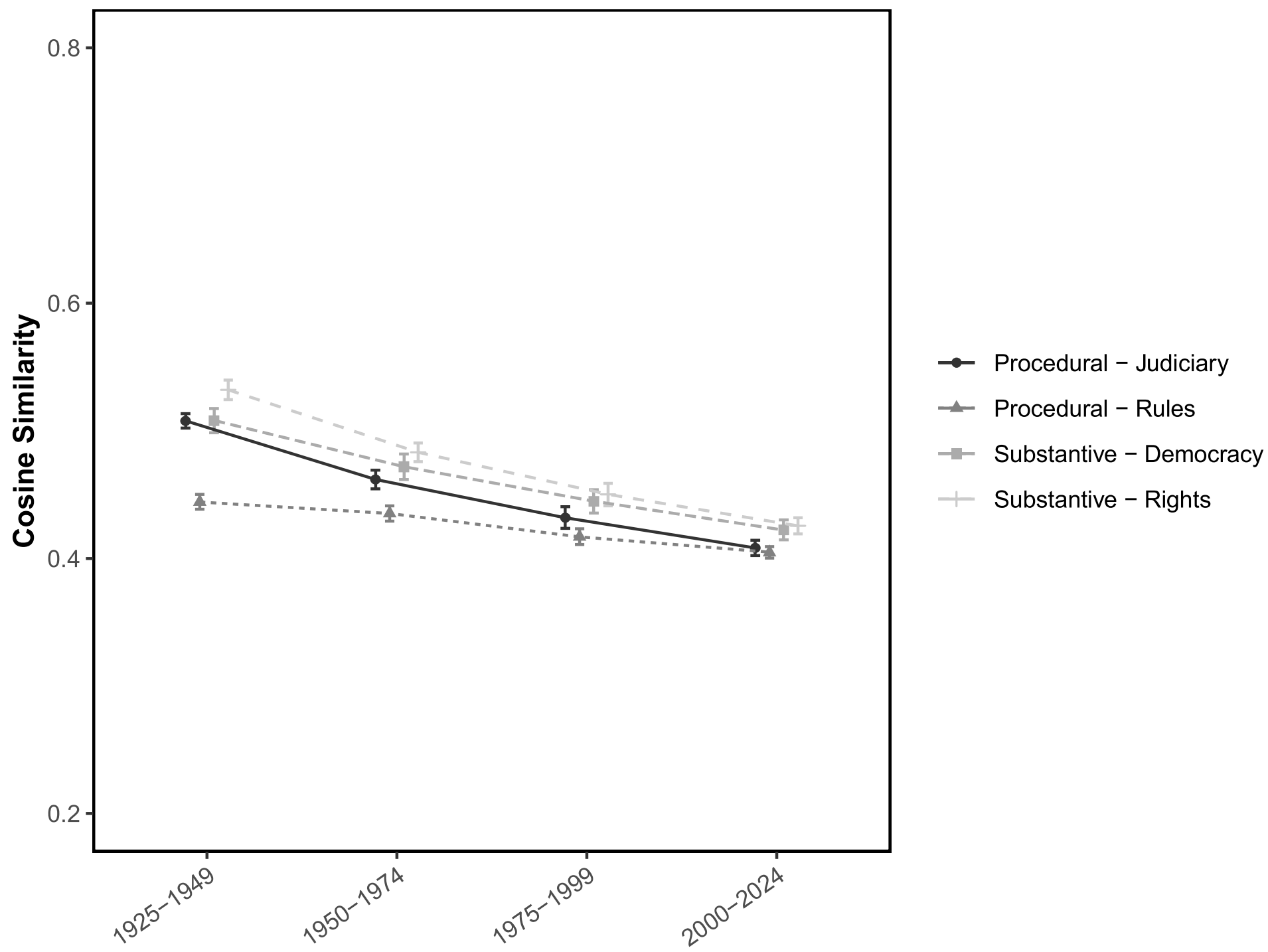

Figure A2: The changing concept of the rule of law in the US (matched corpus) – Alternative chronologically trained models

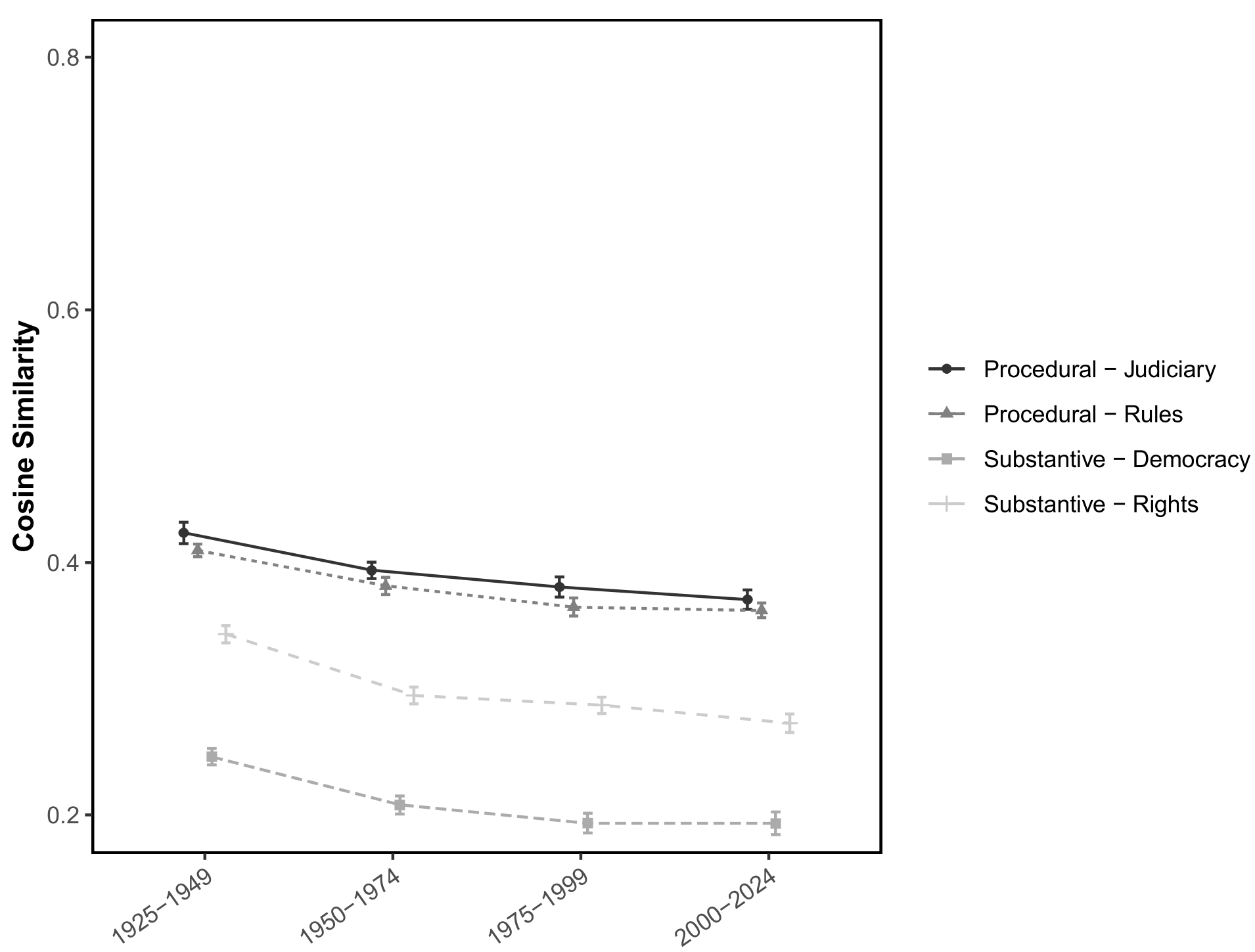

Figure A3: The changing concept of the rule of law in the US (entire corpus) – Alternative chronologically trained models

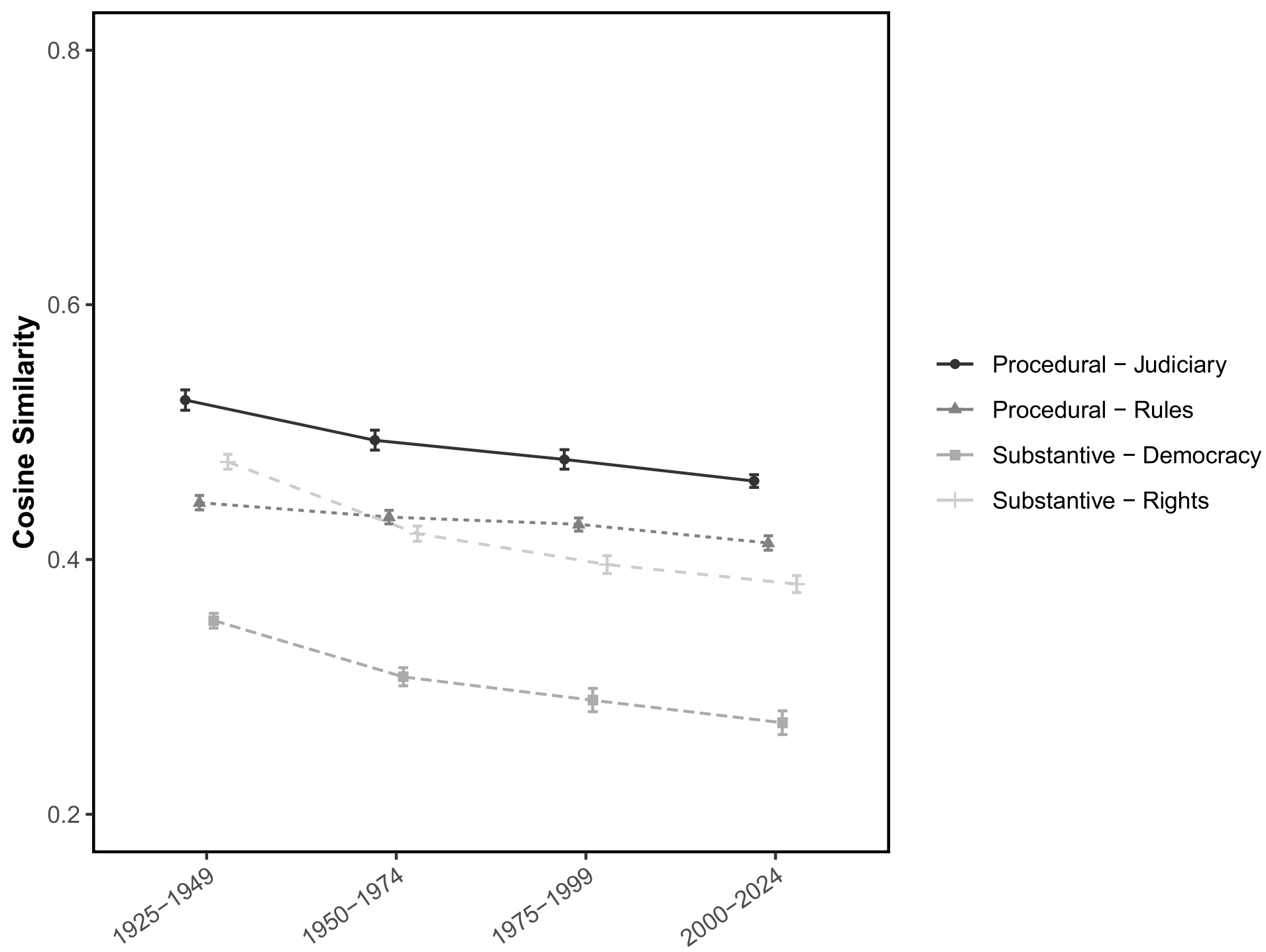

Figure A4: The changing concept of the rule of law in the UK – DistilBERT embeddings

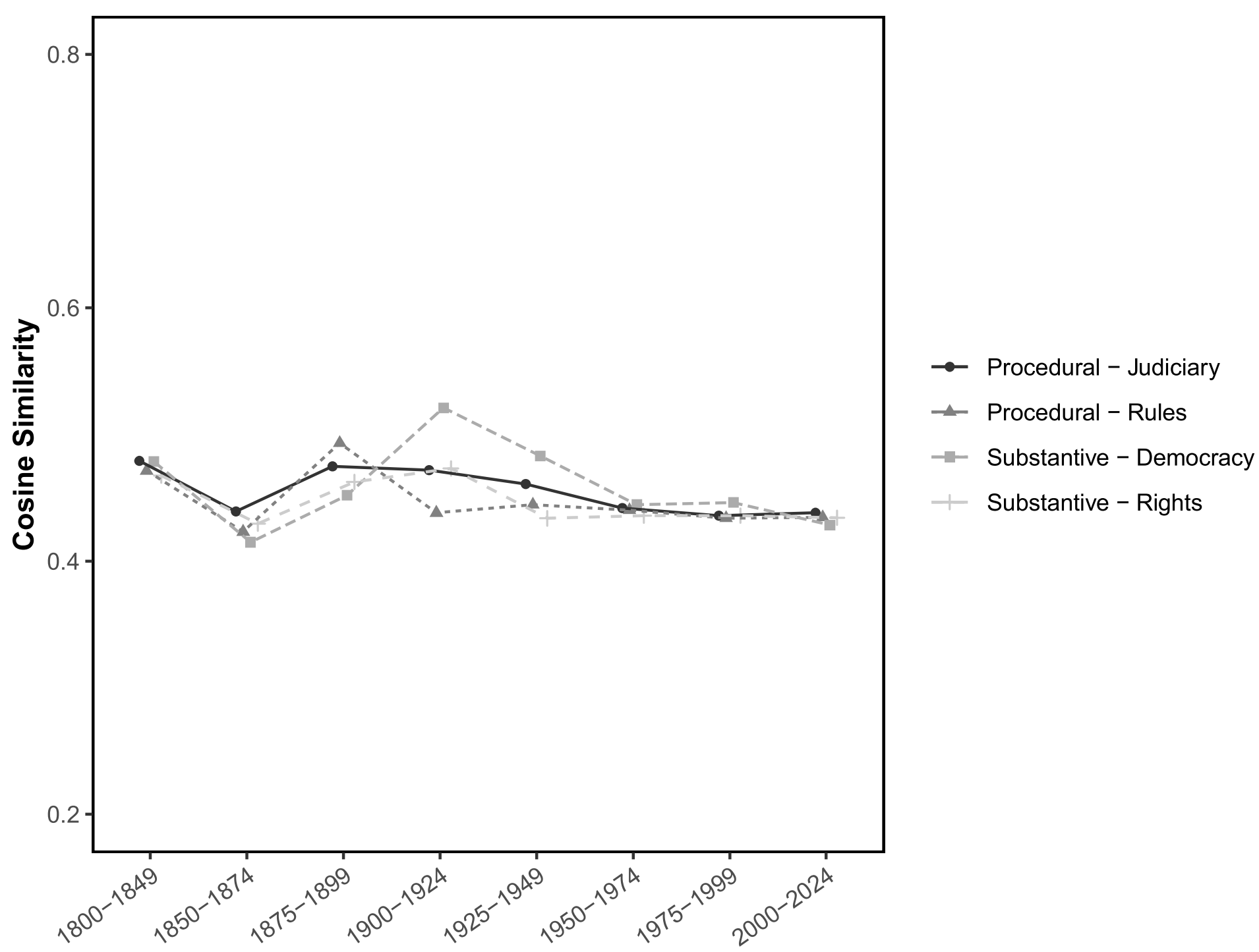

Figure A5: The changing concept of the rule of law in the US – DistilBERT embeddings

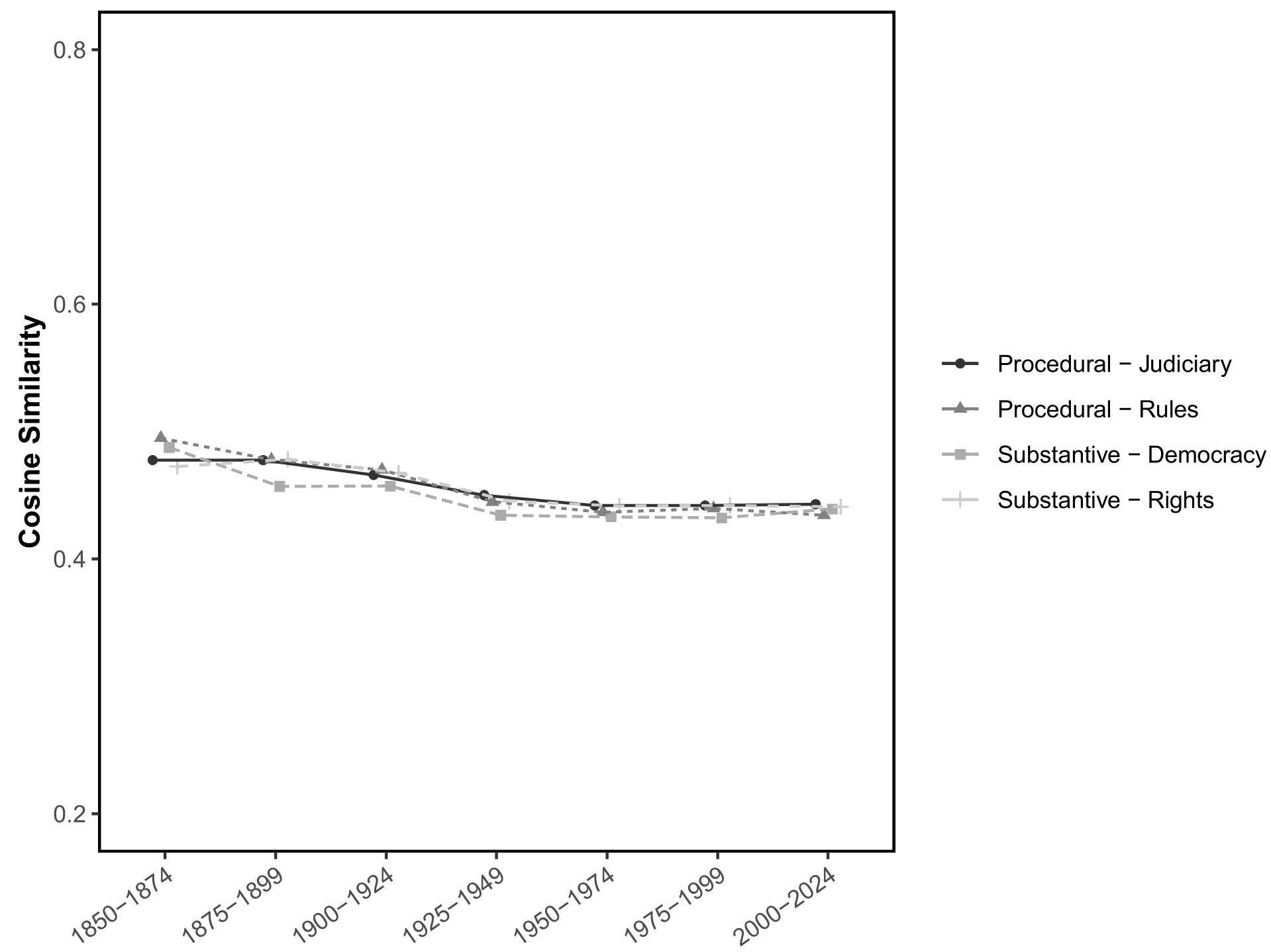

Note. Cosine similarity estimates with 99.5% of the corpus. DistilBERT could not be applied to a small number of text batches because of encoding problems.

Figure A6: The changing concept of the rule of law in the UK – Alternative list of keywords

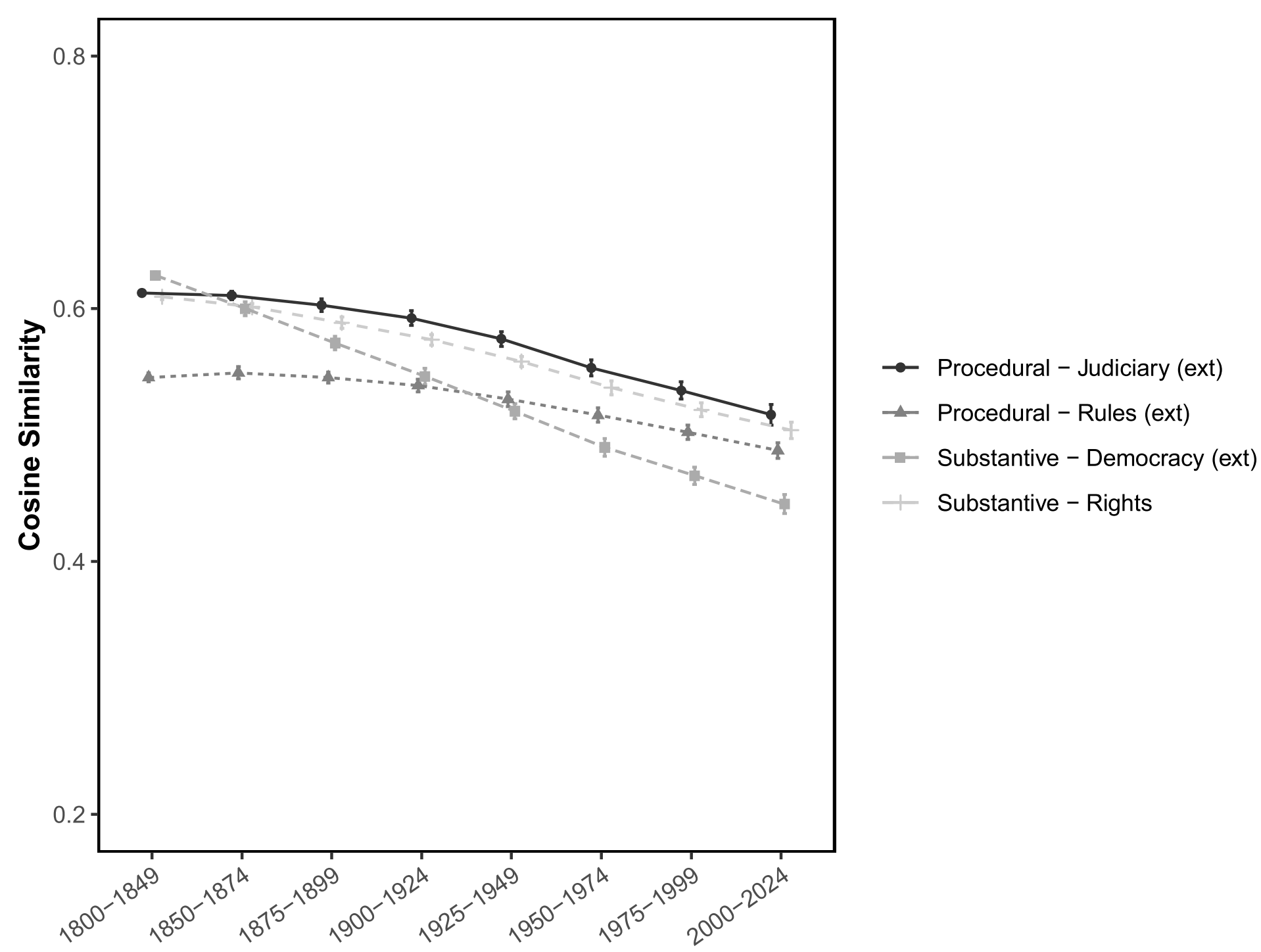

Figure A7: The changing concept of the rule of law in the US (matched corpus) -– Alternative list of keywords

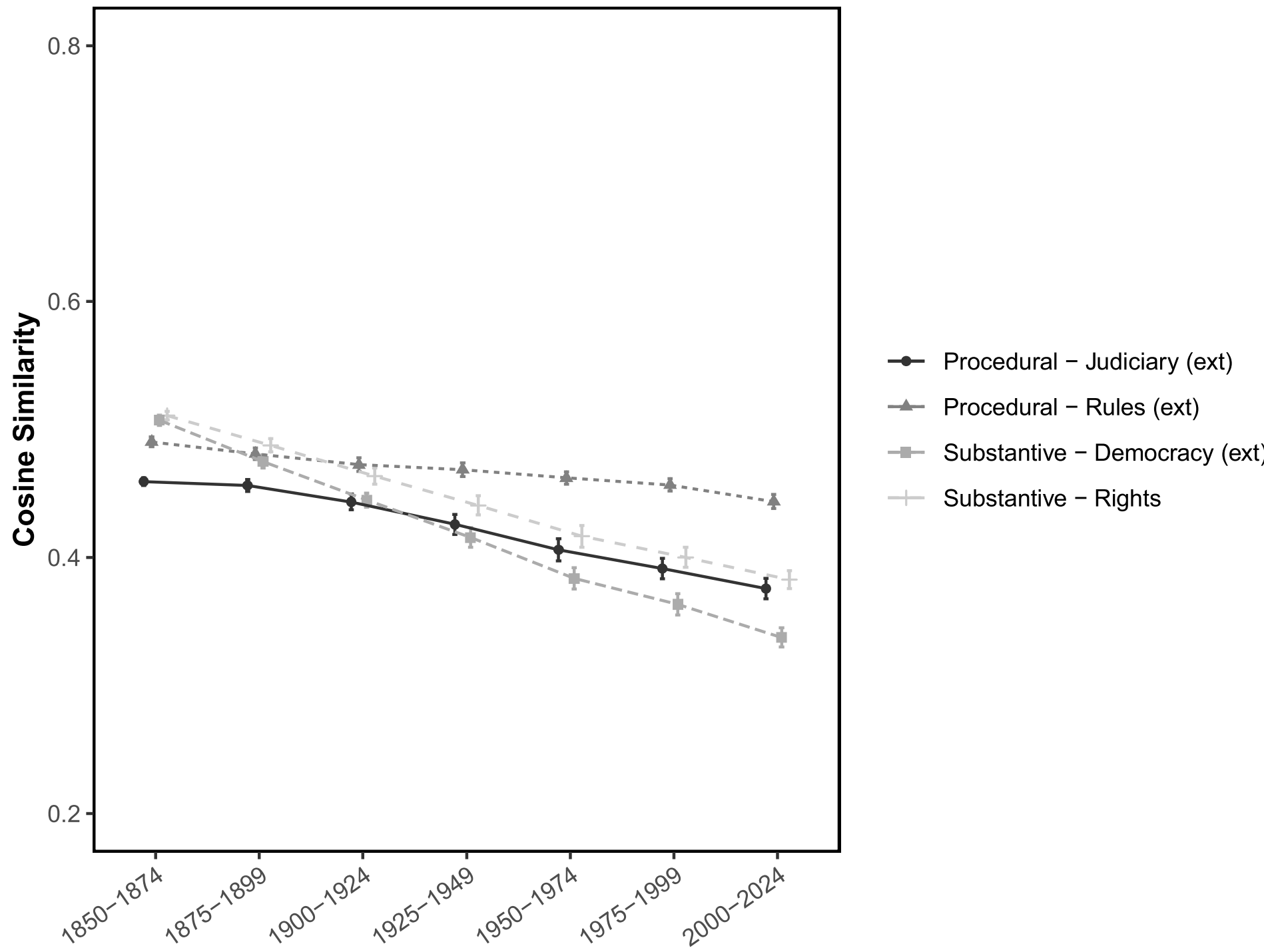

Figure A8: The changing concept of the rule of law in the US (entire corpus) — Alternative list of keywords

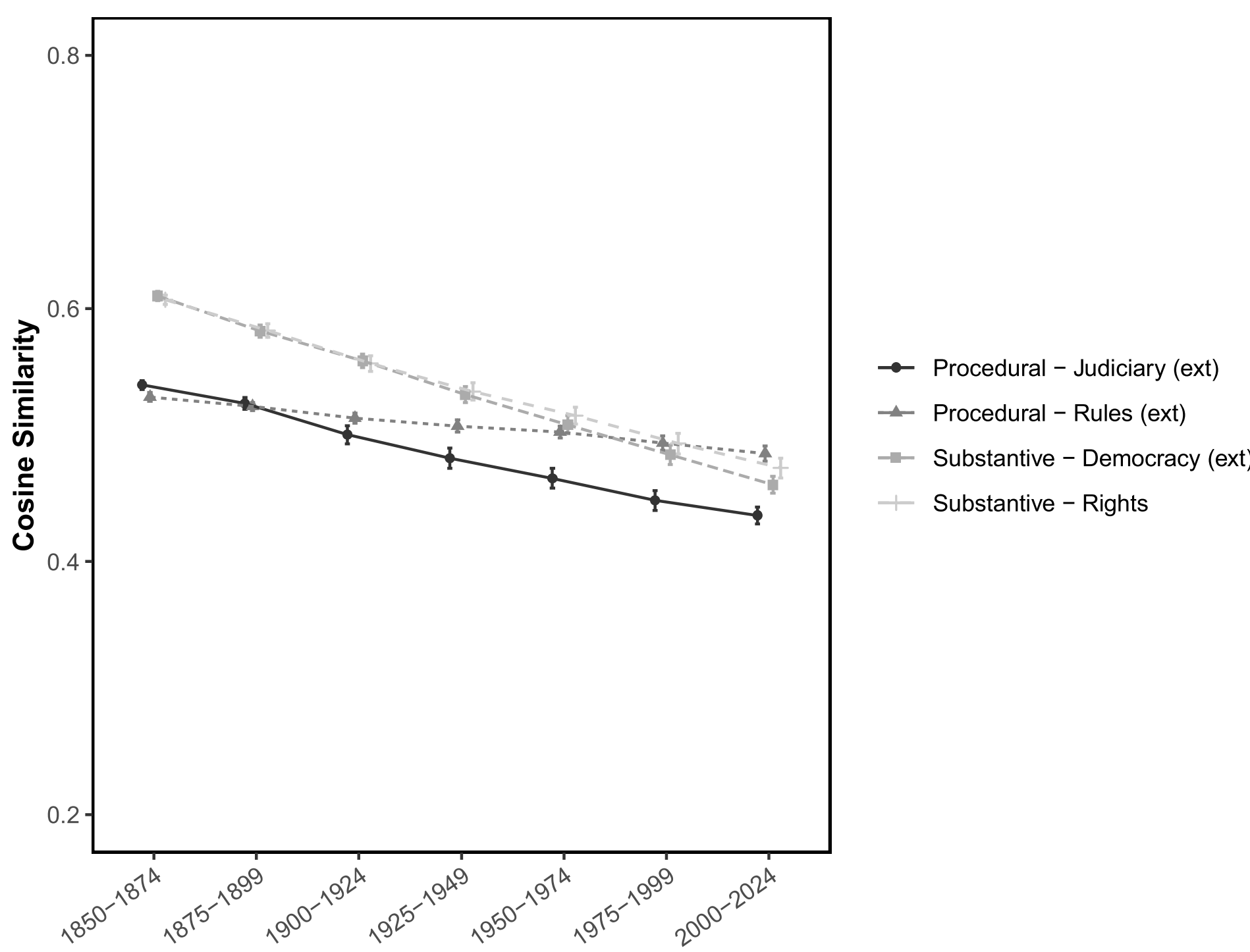

Supplement: Supplementary file 1 [file 10506_2025_9468_MOESM1_ESM.pdf]
